# A Functional Dissection of PTEN N-Terminus: Implications in PTEN Subcellular Targeting and Tumor Suppressor Activity

**DOI:** 10.1371/journal.pone.0119287

**Published:** 2015-04-15

**Authors:** Anabel Gil, Isabel Rodríguez-Escudero, Miriam Stumpf, María Molina, Víctor J. Cid, Rafael Pulido

**Affiliations:** 1 Centro de Investigación Príncipe Felipe, Valencia, Spain; 2 Departamento de Microbiología II, Facultad de Farmacia, Universidad Complutense de Madrid, and Instituto Ramón y Cajal de Investigaciones Sanitarias (IRYCIS), Madrid, Spain; 3 Hubrecht Institute, Utrecht, The Netherlands; 4 BioCruces Health Research Institute, Barakaldo, Spain; 5 IKERBASQUE, Basque Foundation for Science, Bilbao, Spain; University of South Florida College of Medicine, UNITED STATES

## Abstract

Spatial regulation of the tumor suppressor PTEN is exerted through alternative plasma membrane, cytoplasmic, and nuclear subcellular locations. The N-terminal region of PTEN is important for the control of PTEN subcellular localization and function. It contains both an active nuclear localization signal (NLS) and an overlapping PIP2-binding motif (PBM) involved in plasma membrane targeting. We report a comprehensive mutational and functional analysis of the PTEN N-terminus, including a panel of tumor-related mutations at this region. Nuclear/cytoplasmic partitioning in mammalian cells and PIP3 phosphatase assays in reconstituted *S*. *cerevisiae* defined categories of PTEN N-terminal mutations with distinct PIP3 phosphatase and nuclear accumulation properties. Noticeably, most tumor-related mutations that lost PIP3 phosphatase activity also displayed impaired nuclear localization. Cell proliferation and soft-agar colony formation analysis in mammalian cells of mutations with distinctive nuclear accumulation and catalytic activity patterns suggested a contribution of both properties to PTEN tumor suppressor activity. Our functional dissection of the PTEN N-terminus provides the basis for a systematic analysis of tumor-related and experimentally engineered PTEN mutations.

## Introduction

The PI3K/PTEN/AKT pathway is involved in the etiology and progression of a wide variety of human tumors, on account of genetic and epigenetic alterations in the genes that govern the pathway, resulting in aberrant expression levels or activity of its components. In the case of PI3K and AKT, these include protein overexpression or hyperactivity, mainly due to gene amplification or gain-of-function mutations [1–3]. In the case of PTEN, about 30% of human tumors display protein downregulation or loss-of-activity, mainly due to total or partial gene loss or gene transcription, decreased mRNA or protein stability, and loss-of-function mutations [4–6]. Remarkably, the genes encoding p110α (*PIK3CA*, α catalytic subunit of PI3K), AKT1, and PTEN, are all targets for germ-line hereditary mutations in patients with PHTS (PTEN Hamartoma Tumor Syndrome) [7]. Thus, the PI3K/PTEN/AKT pathway has arisen as a major axis for therapeutic intervention in both sporadic and hereditary cancer [8].

The major role of PTEN as a potent tumor suppressor relies on its function as a lipid phosphatase, dephosphorylating the 3’ position of phosphatidylinositol 3,4,5-trisphosphate (PIP3) to generate phosphatidylinositol 4,5-bisphosphate (PIP2), which directly antagonizes the oncogenic activity of PI3K [9,10]. The N-terminal portion of PTEN, together with the C-terminal phosphorylatable PTEN region, is crucial to exert its PIP3 phosphatase activity in cells, being involved in the dynamic regulation of the PTEN open/closed conformational status, which dictates PTEN subcellular localization, stability, and function [11–17]. C-terminal cleavage of PTEN by caspase-3 triggers PTEN nuclear accumulation in a manner dependent on Ran GTPase and a PTEN N-terminal nuclear localization signal (NLS; residues 8–32) [18,19]. PTEN N-terminus also harbours a PIP2-binding motif (PBM; residues 6–15) that mediates binding to membranes, as well as allosteric activation of the enzyme [20–23]. Interestingly, a cytoplasmic localization signal has also been described within this region (residues 19–25) [24]. Nuclear PTEN regulates genome stability, DNA repair, cell cycle, gene expression, and apoptosis [6,25–27], and the loss of PTEN nuclear localization associates in several cancers with tumour progression and poor clinical outcome [28,29]. However, most of PTEN nuclear functions rely on protein-protein interactions rather than on catalysis. Remarkably, monoubiquitylation of Lys13 at the NLS motif is required for PTEN nuclear entry [30], as well as for PTEN secretion via exosomes, a novel PTEN export pathway [31]. In addition, a novel PTEN isoform (PTEN-Long, PTENα; PTEN-L) has been discovered that displays an extended N-terminus. This longer PTEN form can be targeted to the mitochondria and can also be secreted and transferred, as a functional enzyme, from donor to acceptor cells, which is relevant for PTEN-mediated tumor suppression and potential PTEN-based antitumor therapies [32–34].

Here, we have performed a comprehensive functional analysis of the PTEN N-terminal region (residues 2 to 43) using both a humanized *S*. *cerevisiae*-based system and human cancer cell lines. We have uncovered the functional properties of tumor-associated mutations targeting the PTEN N-terminus, and functionally dissected this important region. Our results illustrate the multifaceted role of the PTEN N-terminus in the regulation of PTEN tumor suppression.

## Materials and Methods

### Cell culture, transfections and plasmids

Simian kidney COS-7 (ATCC CRL-1651) cells were grown in DMEM containing high glucose supplemented with 5% heat-inactivated fetal bovine serum (FBS), 1 mM L-glutamine, 100 U/ml penicillin, and 0.1 mg/ml streptomycin. Human osteosarcoma U2OS-derived UTA6 Tet-Off cell line containing Tet-Off plasmid was provided by R. Farràs [35]. U2OS Tet-Off stable cells were grown in the same medium as COS-7 cells, supplemented with 10% FBS, 200 μg/ml geneticin (Invitrogen) and 100 μg/ml hygromycin (Sigma). Cells were grown at 37°C, 5% CO_2_. COS-7 cells were transfected by the DEAE-dextran method and processed after 48 h. To generate stable cell lines overexpressing ectopic PTEN wild type and mutations, U2OS Tet-Off cell line was transfected with pTRE2hyg plasmids using the calcium phosphate method, and pooled clones were selected in the presence of 100 μg/ml of hygromycin. The *Saccharomyces cerevisiae* strain YPH499 (*MATa ade2-101 trp1-63 leu2-1 ura3-52 his3-Δ200 lys2-801)* was used for heterologous expression of mammalian proteins. YPH499 yeast cells were grown in synthetic complete (SC) medium, containing 0.17% yeast nitrogen base without amino acids, 0.5% ammonium sulfate supplemented with appropriate amino acids and nucleic acid bases, and added 2% glucose (SD), galactose (SG) or raffinose (SR), as required. Yeasts were transformed by standard procedures, and drop growth assays were performed as described [36]. pRK5 PTEN constructs have been previously described [18]. The PTEN N-terminal amino acid substitution mutations were made by PCR oligonucleotide site-directed mutagenesis, and mutations were confirmed by DNA sequencing. pTRE2hyg PTEN plasmids were made by subcloning into pTRE2hyg of PCR-obtained PTEN cDNAs from the corresponding pRK5 constructs. YCpLG myc-p110α-CAAX and pYES2 PTEN plasmids have been described [36]. Cloning of PTEN mutations into pYES2 was made from the corresponding pRK5 PTEN mammalian expression vectors.

### Immunofluorescence and microscopy techniques

**Fig 1:**
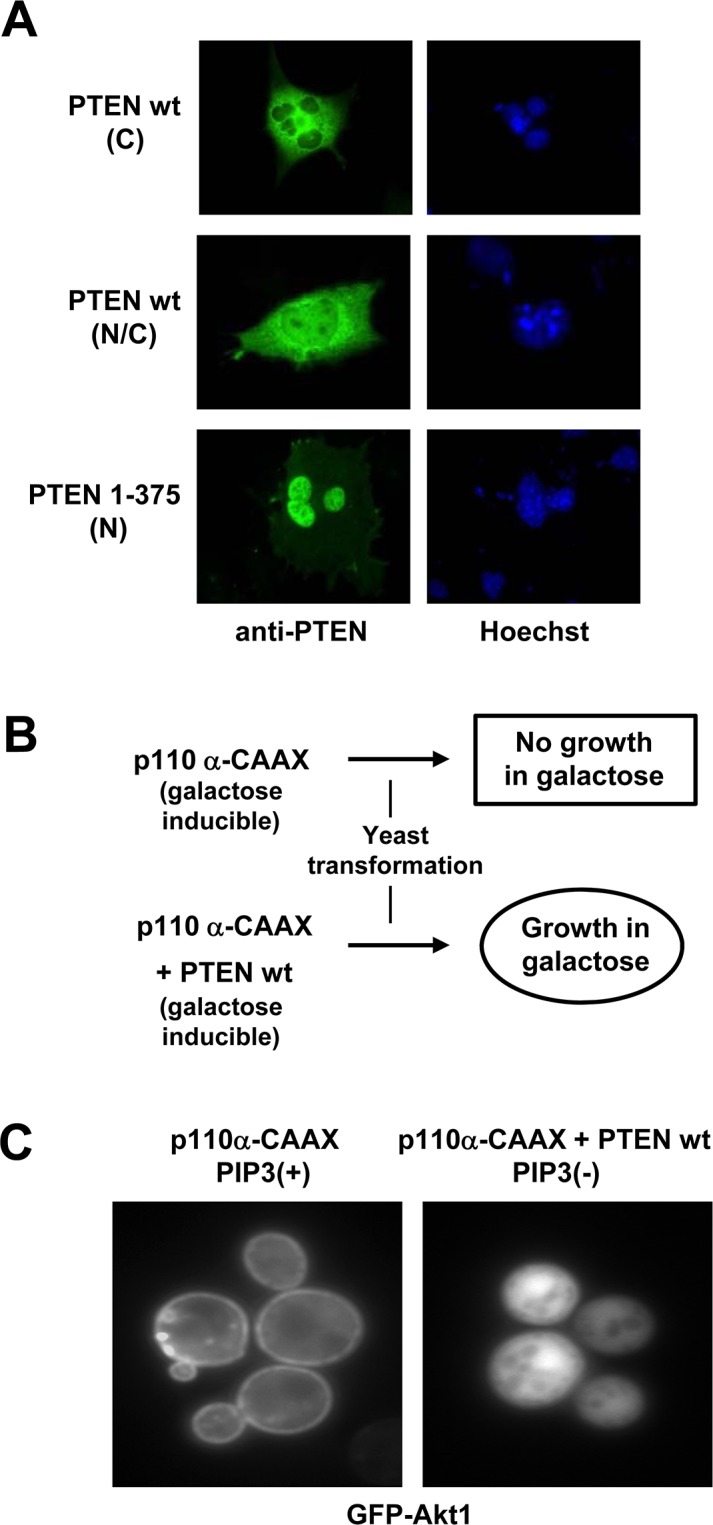
Depiction of the experimental systems used in this study (A) Subcellular localization of PTEN in COS-7 cells. Ectopically expressed PTEN wild type (wt) mainly localizes in the cytoplasm (C) of COS-7 cells (60–70% of transfected cells; upper panels), and some cells show nuclear/cytoplasmic (N/C) localization (30–40% of transfected cells; middle panels). Cells were transfected with plasmids containing the indicated PTEN forms, and subjected to immunofluorescence using anti-PTEN antibodies (green) or Hoechst nuclear staining (blue). By contrast, PTEN 1–375 accumulates in the nucleus of COS-7 cells (80% of transfected cells; lower panels). This feature was used to test the influence of amino acid substitution mutations on PTEN nuclear accumulation. **(B)** Schematic showing the basis of the PTEN PIP3 functional assay in the yeast *S*. *cerevisiae*. Yeast cells expressing p110α-CAAX (a constitutive active form of the p110α catalytic subunit of mammalian PI3K under the control of *GAL1* promoter) do not grow, and this can be reverted by expressing PTEN wild type. This feature was used to indirectly measure PTEN phosphatase activity, by reconstituting yeast viability. **(C)** Indirect measurement of PIP3 hydrolysis by PTEN in yeast is visualized by GFP-Akt1 reporter distribution. In the presence of p110α-CAAX alone (left panel), the GFP-Akt1 reported distributes in the plasma membrane as a result of PIP3 acccumulation [PIP3(+)], whereas in the presence of p110α-CAAX + PTEN wt, GFP-Akt1 displays a diffuse cytoplasmic distribution as a result of PIP3 hydrolysis [PIP3(-)].

To monitor PTEN subcellular location in mammalian cells, immunofluorescence was performed as previously described, using mouse monoclonal anti-PTEN 425A and fluorescein-conjugated anti-mouse antibody [18,37]. For standard microscopy, a Zeiss fluorescence microscope (Thornwood, NY) was used. For confocal microscopy, a Leica confocal microscope (TCS-SP2-AOBS, Mannheim, Germany) was used. For quantitation of PTEN subcellular distribution, at least 100 positive cells were scored for each experiment. Cells were rated as showing nuclear staining (N), cytoplasmic staining (C), or staining within both the nucleus and the cytoplasm (N/C), as illustrated in Fig 1A. Nuclei were identified by Hoescht 33258 (Molecular Probes, Eugene, OR) staining. All pictures were taken under a 400 X magnification. Measurement of green fluorescent protein (GFP)-Akt1 plasma membrane localization in yeast, as an indirect indicator of cellular PIP3 levels, was performed by fluorescence microscopy, as described [36]. ≥150 cells were examined and scored for each condition or experiment for either cytoplasmic or membrane-associated localization, as illustrated in Fig 1C. Cells were examined under an Eclipse TE2000Umicroscope (Nikon) and digital images were acquired with Orca C4742-95-12ER charge-coupled device camera and HCImage software (Hamamatsu).

### Immunoblot

Whole cell protein extracts from U2OS cells overexpressing ectopic PTEN and mutations were prepared by cell lysis in ice-cold lysis buffer (50 mM Tris-HCl, pH 7.5, 150 mM NaCl, 1% IGEPAL CO-630 (Nonidet P-40), 2 mM Na_3_VO_4_, 100 mM NaF, 1mM PMSF, 1 μg/ml of aprotinine, 20 mM Na_4_P_2_O_7_), followed by centrifugation at 15200 g for 10 min and collection of the supernatant. Yeast cell extracts were obtained by standard procedures. Proteins (50–100 μg in the case of mammalian cells; 20–50 μg in the case of yeast) were resolved in 10% SDS-PAGE under reducing conditions and transferred to PVDF membranes. Immunoblot was performed using anti-phospho-Ser473-Akt + anti-phospho-Thr308-Akt and anti-Akt antibodies (Cell Signaling Technologies), anti-PTEN 425A mAb (Andrés-Pons et al., 2005) or rabbit polyclonal anti-PTEN antibodies (Upstate), anti-GAPDH (Santa Cruz Technology) or anti-actin C4 (MP Biomedicals, France) antibodies, followed by horseradish peroxidase (HRP)-conjugated anti-rabbit or anti-mouse (Calbiochem) antibodies. For determination of phospho-Akt content, bands were quantified using ImageQuantTL software (Amersham Biosciences).

### Cell growth assays

Cell growth of U2OS cells was measured as described [38]. For soft-agar growth assays, U2OS Tet-Off cells were plated at a density of 15000 cells per well (12-well plates) in 2 ml of medium with 20% FBS and 0.7% cell culture-tested agar (Sigma), onto the solidified bottom layer of 1.4% agar. Colonies were stained after 3–4 weeks with 0.05% crystal violet. For cell proliferation assays, the 3-[4,5-Dimethylthiazol-2-yl]-2,5-dephenyltetrazolium bromide assay (MTT) was used according to the manufacturer’s protocol (Roche Applied Science). Cells were plated at a density of 2500 cells per well (96-well plates) with complete medium, and cell proliferation was determined at 1–5 days. Yeast cell growth was monitored on solid media by serial dilution-drop growth assays, as described [36].

## Results

### Functional analysis of tumor-associated N-terminal PTEN mutations

In this study, we have monitored PTEN subcellular distribution and PIP3 phosphatase activity using in parallel mammalian and yeast cell systems (Fig 1). When overexpressed in COS-7 cells, wild type PTEN displays a cytosolic or a nuclear/cytosolic distribution; in contrast, the PTEN 1–375 truncation, which mimics a PTEN product of caspase-3 cleavage, accumulates in the nucleus in an N-terminus-NLS-dependent manner (Fig 1A) [18,19]. Heterologous galactose-inducible expression of p110α-CAAX (constitutively active, prenylatable form of the p110α catalytic subunit of PI3K) results in high toxicity in *S*. *cerevisiae* yeast cells as a result of the depletion of essential PIP2 pools. Co-expression of wild type PTEN restores PIP2 levels and suppresses PI3K toxicity. Conversion of PIP3 to PIP2 by PTEN can be monitored either by the suppression of PI3K-dependent yeast growth inhibition (Fig 1B) or by the relocation of a heterologous Akt reporter from the plasma membrane (Fig 1C). This allows for rapid and reliable assessment of PTEN activity *in vivo* [36,39,40].

**Fig 2:**
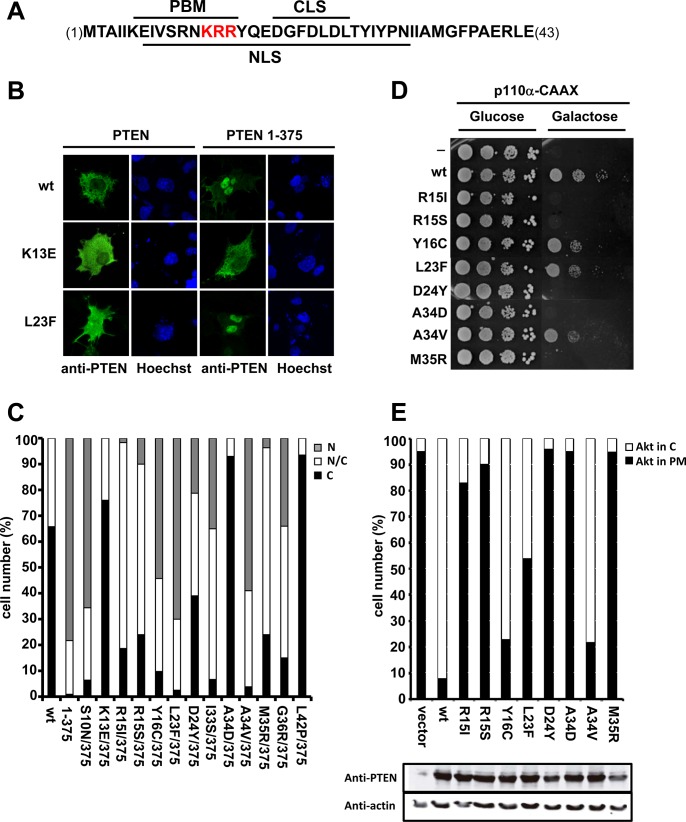
Subcellular localization and functional analysis of tumor-associated N-terminal PTEN mutations. (**A**) Amino acid sequence of PTEN N-terminus (residues 1–43). The nuclear localization signal (NLS), the PIP2-binding motif (PBM), and the cytoplasmic localization signal (CLS) are indicated. The KRR motif (residues 13–15) is in red. (**B, C**) The influence of tumor-associated PTEN mutations on PTEN subcellular localization was assessed by immunofluoresecence on mammalian COS-7 cells. In B, COS-7 cells transfected with wild type (wt; 1–403) or mutated PTEN (1–375, or mutations in a 1–375 background) were analysed by standard immunofluorescence microscopy using anti-PTEN 425A mAb (green). Nuclei were stained with Hoechst (blue). In C, the nuclear/cytoplasmic distribution of PTEN mutations was scored from COS-7 cells processed as in B; N, nuclear localization; C, cytoplasmic localization; N/C, nuclear/cytoplasmic localization. (**D, E**). The influence of tumor-associated PTEN mutations in the *in vivo* PTEN PIP3 phosphatase activity was assessed in yeast. In D, *S*. *cerevisiae* drop growth experiments were performed on co-transformants expressing p110α-CAAX and the indicated PTEN mutations, under repression (Glucose) or induction (Galactose) of the expression of the heterologous mammalian proteins. Expression of p110α-CAAX results in high toxicity in yeast cells as a result of the depletion of essential PIP2, which is converted to PIP3. Co-expression of wild type PTEN hydrolyses PIP3 and restores PIP2 levels and viability. In E, PIP3 hydrolysis by PTEN in yeast cells was monitored indirectly using a GFP-Akt1 reporter protein. Results are shown as percentage of yeast cells that display GFP-Akt1 at the plasma membrane (PM). The bottom panel shows the equivalent expression in the yeast of all PTEN mutations, as assessed by immunoblot using anti-PTEN and anti-actin (as a loading control) antibodies.

The PTEN N-terminal region contains motifs important for PTEN subcellular localization and function (Fig 2A). To analyze the contribution of this region to PTEN tumor suppressor function *in vivo*, we performed a functional analysis of a panel of tumor-associated N-terminal PTEN mutations (COSMIC database; http://cancer.sanger.ac.uk/cosmic) (Fig 2; Table 1; Table 2). The nuclear/cytosolic localization of the mutations was tested in mammalian COS-7 cells. On a PTEN wild type background (1–403), which mostly displays cytoplasmic localization, the mutations L23F, M35R, and G36R showed increased nuclear localization (Fig 2B; Table 1). In contrast, on a PTEN 1–375 background, a different pattern of PTEN nuclear/cytosolic partition in such mutations was observed (Fig 2B and 2C; Table 2). The mutations K13E, A34D, and L42P, fully abrogated the nuclear accumulation of PTEN 1–375. A partial inhibition of nuclear accumulation was observed with the R15I, R15S, D24Y, I33S, M35R, and G36R mutations, whereas the nuclear/cytoplasmic distribution of S10N, Y16C, L23F, and A34V mutations was not significantly altered. It is likely that changes in both nuclear active shuttling and binding to plasma membrane account for the different subcellular distribution patterns of these PTEN mutations.

**Table 1:** Subcellular localization of tumor-associated PTEN mutations motif on a PTEN 1–403 background.

Subcellular localization (%)*			
Mutation	N	C	N/C
wt	0	67	33
S10N	0	72	28
K13E	0	70	30
R15I	0	68	32
R15S	0	92	8
Y16C	2	94	4
L23F	8	25	67
D24Y	0	61	39
I33S	3	70	27
A34D	0	83	17
A34V	0	74	26
M35R	34	2	64
G36R	47	7	46
L42P	0	88	12

*Percentage of COS-7 cells showing nuclear (N), cytoplasmic (C), or nuclear/cytoplasmic staining (N/C) is indicated. Note that, in the context of PTEN wild type (wt) 1–403, most of the mutations displayed predominant cytoplasmic localization. The mutations L23F, M35R, and G36R favored PTEN 1–403 nuclear accumulation. Mutations are from COSMIC database (http://cancer.sanger.ac.uk/cosmic)

**Table 2:** PIP3 functional analysis and subcellular localization of tumor-associated PTEN mutations.

Mutation	PIP3 in vivo activity*	Subcellular localization^†^	Tumor type/disease^#^
wt 1–403	+	C	
wt 1–375	+	N	
S10N	+/-	N	LC,NHML,EC
K13E	-	C	*CS*,Glb,EC
R15I	-	N/C	Glb,EC
R15S	-	N/C	*CS*,*ASD*,Glb,CC
Y16C	+/-	N	Glb,NHML
L23F	+/-	N	Glb
D24Y	-	N/C or C	*BRR*,*CS*,EC,OC,BD
I33S	-	N/C	Glb,EC
A34D	-	C	*BRR*,EC
A34V	+/-	N	EC
M35R	-	N/C	*JPS*,Glb
G36R	-	N/C	*CS*,*LDD*,Glb,SS
L42P	-	C	Glb

* PIP3 *in vivo* activity in yeast was monitored by the reconstitution of the p110α-CAAX-induced lack-of-growth phenotype (+, reconstitution; +/-, partial reconstitution;-, no reconstitution). Data of mutations are on a PTEN 1–403 background, from Fig 2 and from [36].

† Major subcellular localization (N, nucleus; C, cytoplasm; N/C, nucleus/cytoplasm) was determined by immunofluorescence on mammalian COS-7 cells. Data of mutations are on a PTEN 1–375 background, from Fig 2.

^#^ From COSMIC (http://cancer.sanger.ac.uk/cosmic) and HGMD (http://www.hgmd.cf.ac.uk) databases. Abbreviations: BD, bladder cancer; BRR, Bannayan-Riley-Ruvalcaba syndrome; CC, colon carcinoma; CS, Cowden syndrome; EC, endometrial cancer; Glb, glioblastoma; JPS, juvenile polyposis syndrome; LC, lung carcinoma; LDD, Lhermitte-Duclos disease; NHML, non-Hodgkin’s malignant lymphoma; OC, ovarian carcinoma; SC, stomach carcinoma; SS, synovial sarcoma. In italic, germ-line mutations.

Next, the PIP3 phosphatase activity of the tumor-associated N-terminal PTEN mutations was assessed using the *S*. *cerevisiae* heterologous reconstitution system. In these experiments, the mutations were tested in a PTEN 1–403 background, and the expression levels of all mutants were similar to the expression levels of wild type PTEN (Fig 2E) suggesting that the tested tumor-associated N-terminal PTEN mutations do not affect PTEN protein stability. As shown in Fig 2D and 2E, and Table 2, the mutations K13E, R15I, R15S, D24Y, I33S, A34D, M35R, G36R, and L42P totally abrogated PTEN activity in the yeast model, whereas mutations S10N, Y16C, L23F, and A34V partially reduced PTEN activity. Interestingly, the latter tumor-associated mutations that did not strongly affect PTEN activity, did not significantly modify the PTEN 1–375 nuclear/cytosolic distribution either, pointing towards an overlapping tumor suppressor role of the PTEN N-terminus for its PIP3 catalytic activity and its nuclear localization *in vivo* (Table 2).

### Functional analysis of PTEN N-terminal region by comprehensive mutagenesis

**Fig 3:**
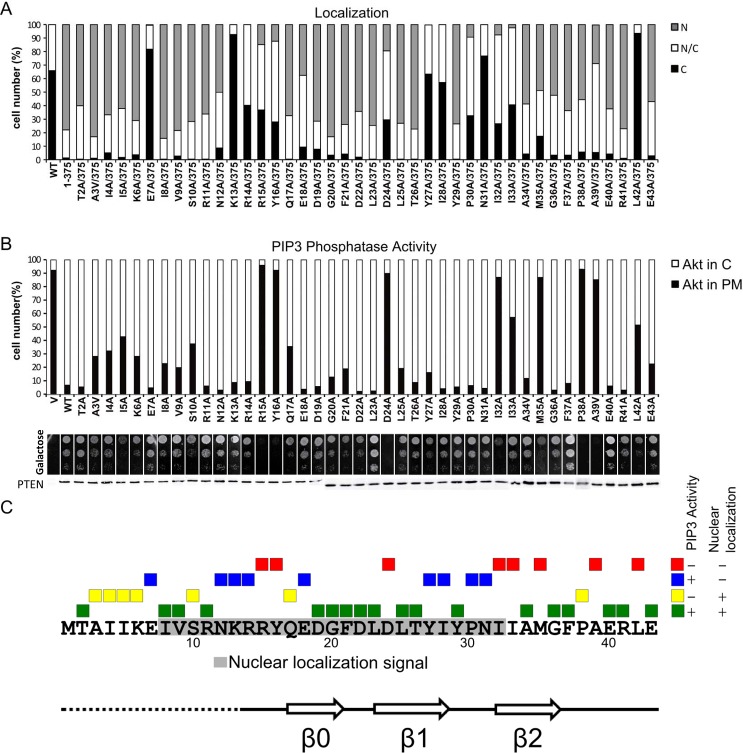
Subcellular localization and functional analysis by Ala-scanning of PTEN N-terminal region reveals distinct subgroups of PTEN mutations. (**A**) Nuclear/cytoplasmic distribution of PTEN N-terminal mutations, monitored as in Fig 2C. Nuclear/cytoplasmic distribution of mutations V9A to E18A is as in [18]. (**B**) Influence of PTEN N-terminal mutations in the *in vivo* PTEN PIP3 phosphatase activity, assessed in yeast. In the upper panel (bars graph), the PIP3 phosphatase activity of PTEN N-terminal mutations was monitored as in Fig 2E. In the middle panel (drop growth), growth was monitored as in Fig 2D. The activity of mutations K6A to E18A is as in [36]. In the bottom panel, the equivalent expression in the yeast of all PTEN mutations, as assessed by immunoblot using anti-PTEN antibodies, is shown. **(C)** Scheme summarizing the Ala-scanning functional results and the distinct subgroups of PTEN mutations. PTEN N-terminal amino acid sequence (residues 1–43) is shown, and the NLS (grey) motif is indicated. The functional consequences of each Ala-substitution are indicated with a colour code: green (+ +), no effect; yellow (- +), normal PIP3 phosphatase activity but impaired nuclear accumulation; blue (+-), impaired phosphatase activity but normal nuclear accumulation; red (—), impaired phosphatase activity and nuclear accumulation. For nuclear localization in the background of PTEN 1–375, we consider positive those mutants that showed over 50% of cells with nuclear localization. For *in vivo* activity on PIP3, we consider positive those mutants that showed under 30% of cells with Akt at the plasma membrane and significantly rescued p110α-induced growth inhibition. The first Met (M) was not mutated, and corresponds to PTEN wild type.

To further dissect the involvement of PTEN N-terminal region in its function, we performed a full Ala-scanning mutagenesis of the PTEN region from residue 2 to residue 43 (Ala residues were mutated to Val). The mutated region spans the N-terminal PTEN tail and the three N-terminal β-strands at the PTP domain (Fig 2A and Fig 3C). Both subcellular localization and yeast reconstitution experiments were performed. Mutations at the basic residues of the N-terminus NLS region, including K13A, R14A, and R15A, as well as mutations E7A, Y16A, D24A, Y27A, I28A, P30A, N31A, I32A, I33A, and L42A, inhibited nuclear entry of PTEN 1–375, whereas mutations N12A, E18A, M35A, and A39V also inhibited nuclear accumulation but to a lesser extent (Fig 3A; and [18]). On the other hand, the cytoplasmic localization of PTEN 1–403 shifted partially to the nucleus in the mutations D19A, G20A, and F21A (Table 3), in consistence with a previously mapped cytoplasmic localization signal (CLS) at these residues [24]. Thus, several regions at the PTEN N-terminus are important for PTEN nuclear localization.

**Table 3:** Subcellular localization of Ala-scanning PTEN mutations on a PTEN 1–403 background.

Subcellular localization (%)*			
Mutation	N	C	N/C
T2A	0	65	35
A3V	0	60	40
I4A	9	71	21
I5A	0	71	30
K6A	0	68	32
E7A	1	82	18
I8A	0	72	28
V9A	0	65	35
S10A	0	69	31
R11A	0	54	46
N12A	0	71	29
K13A	0	90	10
R14A	0	81	19
R15A	0	74	26
Y16A	0	77	23
Q17A	0	80	20
E18A	0	75	25
D19A	17	12	71
G20A	47	3	50
F21A	61	2	37
D22A	9	66	25
L23A	0	61	39
D24A	6	75	19
L25A	2	64	34
T26A	0	77	23
Y27A	0	57	44
I28A	0	65	35
Y29A	0	64	36
P30A	0	68	33
N31A	0	50	50
I32A	0	63	37
I33A	3	70	27
A34V	0	74	26
M35A	0	70	30
G36A	0	64	36
F37A	0	69	31
P38A	1	70	29
A39V	0	55	45
E40A	0	65	35
R41A	0	70	30
L42A	0	78	22
E43A	0	71	29

*Percentage of COS-7 cells showing nuclear (N), cytoplasmic (C), or nuclear/cytoplasmic staining (N/C) is indicated. Note that, in the context of PTEN 1–403, most of the mutations did not affect the localization of PTEN wild type (wt), with the exception of some mutations in the cytoplasmic localization signal at residues 19–25, which favored PTEN 1–403 nuclear accumulation

Regarding the PIP3 phosphatase activity of the PTEN mutations in the yeast *in vivo* assay, a set of mutations, including R15A, Y16A, D24A, I32A, M35A, P38A, and A39V, displayed complete loss-of-function, whereas the rest of the mutations partially compromised (A3V, I4A, I5A, K6A, S10A, Q17A, I33A, and L42A) or did not considerably affect PTEN activity on PIP3 in yeast (Fig 3B). The expression of all mutant versions was similar to the expression of wild type PTEN (Fig 3B). Therefore, many of the mutations differentially affected PTEN nuclear accumulation and PTEN phosphatase activity *in vivo*, suggesting a distinctive control of these two processes. Also, mutations R15A, Y16A, D24A, I32A, I33A, M35A, A39V, and L42A abrogated both nuclear accumulation and PIP3 phosphatase activity of PTEN, indicating that the relationship between these two properties extends beyond the NLS- and the PBM-residues (Fig 3C). Together, these results provide a map of the contribution of particular residues at distinct PTEN N-terminal regions to both subcellular localization and catalytic activity *in vivo*. PTEN N-terminal mutations can thus be categorized in three sub-groups: a group affecting PIP3 phosphatase activity, a group involved in nuclear accumulation, and a third group involved in both phosphatase activity and nuclear localization.

### Differential involvement of residues at the PTEN N-terminal KRR motif in PTEN phosphatase activity and subcellular localization

**Fig 4:**
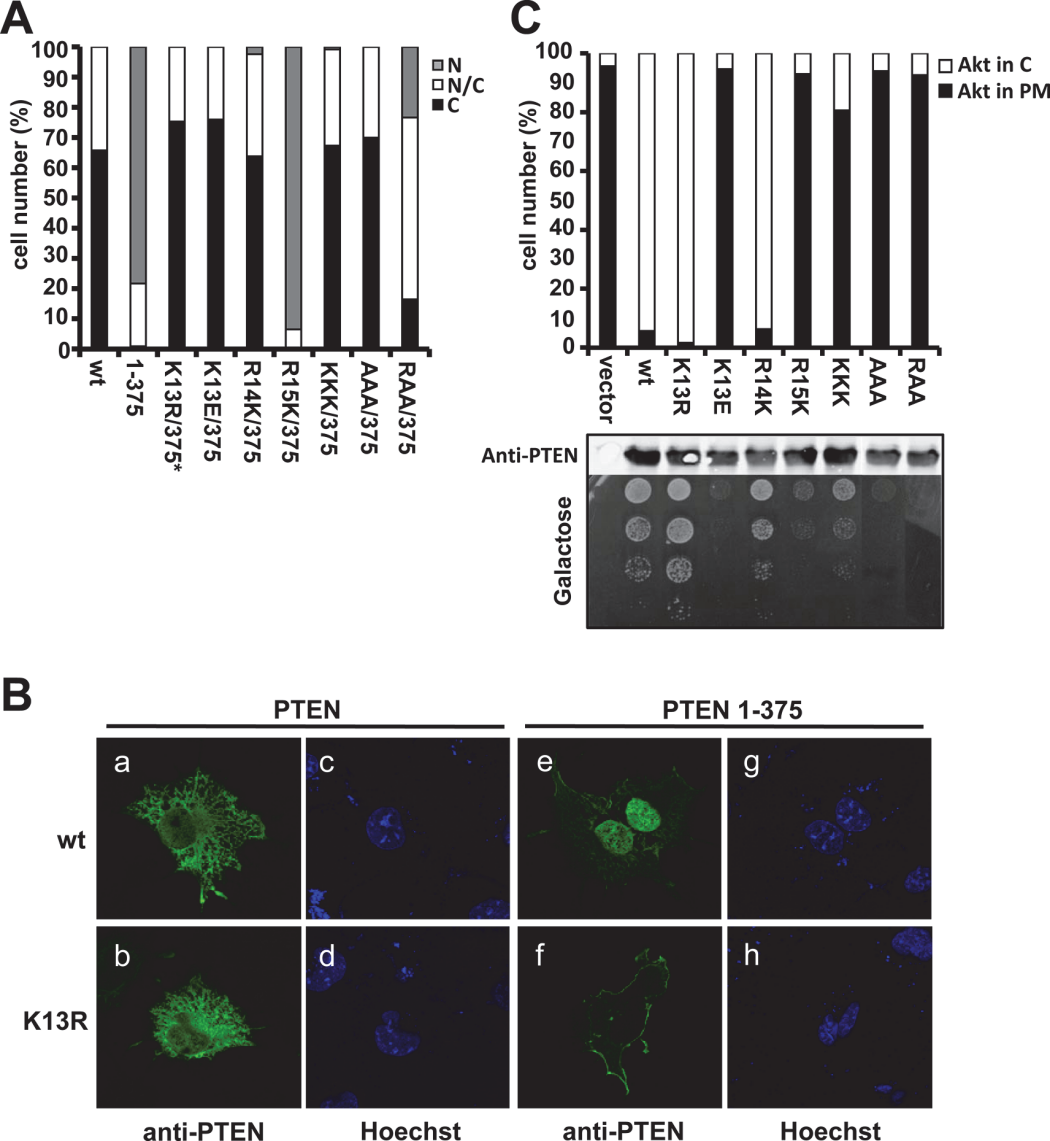
Subcellular localization and functional analysis of PTEN mutations at the N-terminal KRR motif. (**A**) Arg13 and Lys14 are essential for nuclear localization. The nuclear/cytoplasmic distribution of PTEN mutations at the KRR motif was monitored by standard immunofluorescence microscopy, as in Fig 2C. *Note that standard microscopy does not provide information on plasma membrane localization for K13R (see B, panel f). KKK, R14K/R15K; AAA, K13A/R14A/R15A; RAA, K13R/R14A/R15A. (**B**) COS-7 cells transfected with PTEN wild type (wt) or mutations were analysed by confocal immunofluorescence microscopy using anti-PTEN 425A mAb (green). Nuclei were stained with Hoechst (blue). Note the plasma membrane staining on the PTEN 1–375 K13R mutation (panel f). (**C**) Arg15 is essential for PIP3 phosphatase activity. The influence of PTEN mutations at the KRR motif in the *in vivo* PTEN PIP3 phosphatase activity was assessed in yeast. In the upper panel (bars graph), the PIP3 phosphatase activity of PTEN mutations at the KRR motif was monitored as in Fig 2E. The middle panel shows the equivalent expression in the yeast of all PTEN mutations, as assessed by immunoblot using anti-PTEN antibodies. In the bottom panel (drop growth), growth was monitored as in Fig 2D.

Since specific residues from the positively charged motif at the PTEN N-terminus NLS (Lys13Arg14Arg15; KRR motif) (Fig 2A) are important for nuclear localization or phosphatase activity *in vivo*, we performed additional amino acid substitutions in this motif aimed to change its charge and/or its bulk. As shown in Fig 4A, charge conservative replacement K13R and R14K mutations, as well as K13E mutation, affected the nuclear localization of PTEN 1–375. It should be mentioned that inhibition of nuclear accumulation on PTEN 1-375/K13R associated with increased plasma membrane localization, as evidenced by confocal microscopy (Fig 4B). On the other hand, mutation R15K did not inhibit PTEN 1–375 nuclear accumulation, and increased the nuclear accumulation of PTEN 1–403 (Fig 4A, Table 4). Thus, Lys13 and Arg14, but not Arg15, are essential for PTEN nuclear accumulation. Combined mutations rendering either a KKK (R14K/R15K) or an AAA (K13A/R14A/R15A) motif were also excluded from the nucleus, whereas the mutation rendering a RAA (K13R/R14A/R15A) motif displayed a partial nuclear accumulation. In contrast to the results obtained for PTEN nuclear accumulation, the R15K mutation did not display phosphatase activity *in vivo*, whereas the activity of the K13R and R14K mutations was not diminished in comparison with wild type PTEN. In fact, the K13R mutation even displayed an increased ability to counteract PI3K activity in the yeast model. Finally, the activity of mutation K13E was totally absent (Fig 4C). The expression of all mutations at the KRR motif was similar to the expression of wild type PTEN (Fig 4C). These results confirm that Arg15, but not Lys13 or Arg14, is essential for the PIP3 phosphatase activity of PTEN *in vivo*. This is in agreement with the finding that Arg15 is critical for targeting PTEN to the plasma membrane [41]. Together, our results reveal a differential and complementary involvement of PTEN residues Lys13, Arg14, and Arg15 in PTEN nuclear localization and phosphatase activity in cells.

**Table 4:** Subcellular localization of PTEN mutations at the N-terminal KRR motif on a PTEN 1–403 background.

Subcellular localization (%)*			
Mutation	N	C	N/C
K13R	8	46	46
K13E	0	75	25
R14K	0	73	27
R15K	44	18	38
KKK	0	72	28
AAA	0	74	26
RAA	0	75	25

*Percentage of COS-7 cells showing nuclear (N), cytoplasmic (C), or nuclear/cytoplasmic staining (N/C) is indicated. KKK, R14K/R15K; AAA, K13A/R14A/R15A; RAA, K13R/R14A/R15A. Note that the mutation R15K favored PTEN 1–403 nuclear accumulation.

### Functional analysis of PTEN N-terminal mutations in mammalian cells

**Fig 5:**
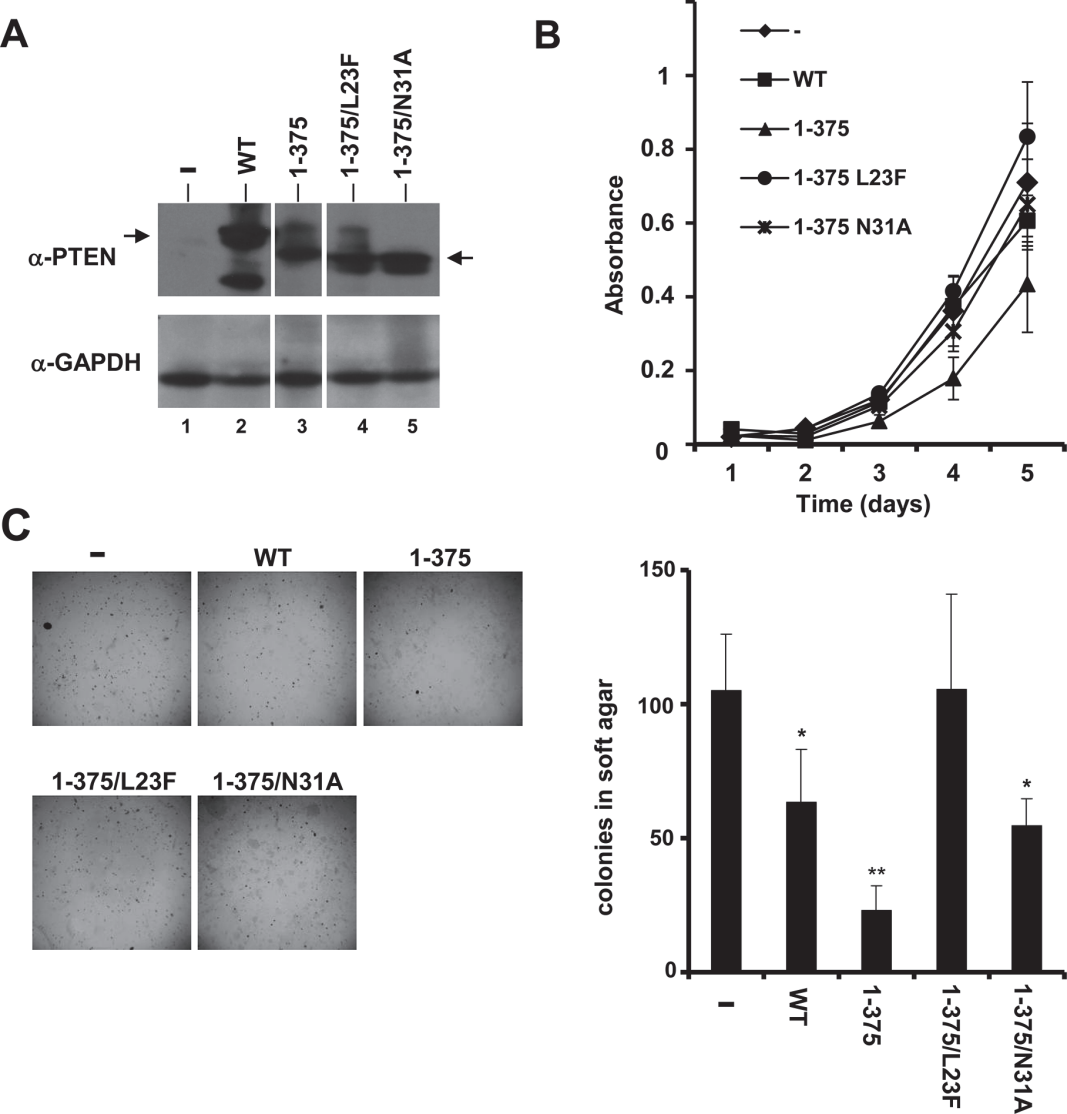
Functional analysis in mammalian cells of PTEN mutations displaying distinctive nuclear accumulation and PIP3 phosphatase activity. (**A**) The ectopic expression of PTEN wild type (WT) and mutations in U2OS clones was monitored by immunoblot with an anti-PTEN antibody (upper panel). The arrows indicate the migration of the full-length (residues 1–403) or the truncated (residues 1–375) recombinant PTEN. Expression of GAPDH is shown in the lower panel as a loading control. The figure shows non-adjacent bands from the same blot. (**B**) Proliferation of the distinct U2OS clones during 5 days of culture. Data are shown as the mean + SD of the absorbance from three experiments, corrected for background.-, empty vector. (**D**) Growth in soft agar of the distinct U2OS clones. Cells were plated on soft agar, and pictures (X40 magnification) were made after 3–4 weeks of growth (upper part). The bottom graph shows the quantification of the number of colonies per plate for each clone. Data are shown as the mean + SD from three independent experiments. ** p<0.001, * p<0.05 with respect to PTEN wild type.-, empty vector.

To test the function on mammalian cells of PTEN N-terminal mutations displaying distinctive nuclear accumulation, stable human osteosarcoma U2OS Tet-Off cell lines expressing PTEN 1-375/L23F and 1-375/N31A mutations were generated, and cell proliferation and soft-agar focus formation assays were performed. As a comparison, cells expressing empty vector, PTEN wild type, or PTEN 1–375 were also analysed (Fig 5A). As shown above, the L23F mutation displays compromised/partial PIP3 catalytic activity in the yeast, without affecting the nuclear accumulation of PTEN 1–375 in mammalian cells. Conversely, the N31A mutation did not affect PIP3 catalytic activity in the yeast, but impaired the nuclear accumulation of PTEN 1–375 in mammalian cells (Fig 2 and Fig 3). Cells expressing PTEN 1–375 grew slower on plastic and formed less colonies in soft agar than cells expressing PTEN wild type. Remarkably, cells expressing PTEN 1-375/L23F behaved in both assays as empty vector cells, indicating a complete loss-of-function of this mutation in mammalian cells in terms of cell growth inhibitory capacity. On the other hand, cells expressing PTEN 1-375/N31A behaved as PTEN wild type cells, displaying better growing properties than PTEN 1/375 cells (Fig 5B and 5C). These results support the notion that partial loss of PIP3 phosphatase activity of PTEN is enough to confer a loss-of-function phenotype in mammalian cells, and suggest that impairment of PTEN nuclear accumulation may affect the full capacity of PTEN to control cell growth.

## Discussion

*PTEN* is the second most frequently mutated tumor suppressor gene in human cancers. Although tumor-associated gross mutations in *PTEN* abrogate all its tumor suppressor functions by means of complete loss of the PTEN protein, the phenotype caused by disease-linked *PTEN* point mutations is more variable. This could be relevant for the implementation of specific therapies and preventive measures to patients and affected families. For instance, most of the germ-line *PTEN* point mutations found in patients with PHTS abrogate PTEN PIP3-phosphatase activity. By contrast, some patients with autism spectrum disorders are also carriers of *PTEN* germ-line point mutations and such variants do not result, in general, in total loss of PTEN PIP3 phosphatase activity [42–44]. In addition to PTEN intrinsic catalysis, other biological properties of PTEN, including protein-protein interactions and subcellular localization, may be determinant for its lipid phosphatase activity and tumor suppression *in vivo*. This makes multifunctional analyses of the PTEN variants found in patients necessary.

The N-terminal region of PTEN contains overlapping NLS/PBM motifs that regulate PTEN subcellular localization and catalytic activity, and this region is targeted by tumor-associated mutations with high incidence (15% of PTEN total mutated samples target residues 1–40; about 40% of these being missense mutations; COSMIC database). Moreover, some PTEN tumor-associated mutations at PTEN N-terminus display a reduced ability to bind to the plasma membrane [45]. Our functional analysis of the PTEN N-terminus illustrates that tumor-associated mutations in this PTEN region are selected for loss-of-function, in terms of PIP3 phosphatase activity in a yeast-based *in vivo* setting and nuclear localization in mammalian cells, indicating the additive relevance of both properties in PTEN-mediated tumor suppression. This is in agreement with the distinct lipid phosphatase-independent tumor suppressor functions ascribed to nuclear PTEN [46–51]. By contrast, tumor-associated mutations targeting other PTEN regions, including the ATP-binding sites, show enhanced nuclear accumulation [52]. The lack of nuclear PTEN in many human cancers has been documented, and proposed as a factor of poor prognosis for some tumor types [27]. It would be interesting to contrast these findings on PTEN nuclear exclusion in human tumors with the analysis of the phosphatase activity of the non-nuclear remnant PTEN protein. The KRR residues 13–15 in the PTEN NLS/PBM, as well as the β2-sheet residues 31–36, are targeted in tumors for loss-of-function mutations (Table 1). The KRR residues are likely to mediate direct interactions with PIP2 or nuclear transporters, required for PTEN catalysis and nuclear accumulation [18,20,23,53]. On the other hand, the PTEN residues at the β2-sheet are highly hydrophobic and not solvent-exposed [54], suggesting that mutations at this region compromise functional PTEN local folding. In this regard, our Ala-scanning functional analysis suggests that the integrity of Lys13, Arg14, and residues 31–36, is more relevant for nuclear accumulation than for PIP3 phosphatase activity *in vivo*. On the other hand, integrity of Lys15 and Tyr16 seems to be more important for PIP3 phosphatase activity than for nuclear accumulation. We have found several functional categories of PTEN mutations in our Ala-scanning analysis, including mutations that did neither affect nuclear accumulation nor PIP3 phosphatase activity (+ +), mutations that compromised both nuclear accumulation and PIP3 phosphatase activity (—), and mutations that discriminated between these two functions (+-) (- +) (Fig 2C). In conclusion, although PTEN-PIP2 binding and PTEN-nuclear transport mechanisms share recognition motifs, our results suggest the existence of specific molecular determinants for each of these two activities on the PTEN N-terminus. In line with this notion, the K13R mutation favored PTEN targeting to the plasma membrane in a nuclear-PTEN-mutation background (PTEN 1-375/K13R), but not in the cytosolic wild type-PTEN background (PTEN K13R). A similar phenomena has been found for other PTEN mutations, including mutations at the PTEN N-terminus, which increased their accumulation at the plasma membrane when combined with a PTEN C-terminal-phosphorylation defective mutation [41,42]. Interestingly, sandwiched between clusters of residues important for nuclear localization at the PTEN N-terminus, a cytoplasmic localization signal has been described at residues 19–25 [24]. Our finding that PTEN mutations in this region partially shift to the nucleus support the idea that it could behave similarly to the nuclear exclusion motifs defined in other PTEN regions [18]. This documents the complexity of PTEN N-terminus as a determinant to drive mechanisms of subcellular targeting.

We have tested in mammalian cells the tumor suppressor capacity of PTEN mutations displaying differential PIP3 phosphatase activity and nuclear accumulation properties. The caspase-3 mimicking PTEN C-terminal truncation, PTEN 1–375, which accumulates in the nucleus, displayed enhanced cell growth inhibition, when compared to PTEN wild type. Since the presence of an intact PTEN C-terminal tail keeps PTEN in a closed inactive conformation, which is neither competent to go to the nucleus nor to the plasma membrane [11,13,14,17], it is difficult to delimitate the contribution of the nuclear accumulation of PTEN 1–375 on its tumor suppressor activity. Our results using PTEN 1-375/L23F and 1-375/N31A mutations indicate that both the PTEN PIP3 phosphatase activity and the PTEN capacity to accumulate in the nucleus are important for PTEN tumor suppression.

Also of interest is our finding that Ala-substitution of residues at the non-crystalized disordered N-terminal PTEN region, including Ala3, Ile4, and Ile5, affects PTEN PIP3 phosphatase activity *in vivo*, suggesting the existence of additional PTEN regulatory elements in this region. This is sustained by the presence of *PTEN* mutations targeting these PTEN N-terminal residues in tumors (COSMIC database). Importantly, residues within the unique region of the PTEN longer isoform, contiguous to the region studied here, are also targeted by mutations in tumors [32], although the intrinsic PIP3 catalytic activity of these mutations is not altered when assessed in the yeast [55]. Further molecular and *in vivo* analyses are necessary to fully understand the regulatory mechanisms imposed by the standard PTEN N-terminus, and by the adjacent residues from the PTEN longer isoform, to PTEN tumor suppressor function in mammalian cells. Our comprehensive functional analysis of PTEN N-terminus provides a foundation to understand and exploit the N-terminal properties of PTEN to intervene therapeutically in PTEN-mediated tumor suppression.
